# Histomorphometric Analysis of Bone Density in Relation to Tactile Sense of the Surgeon During Dental Implant Placement

**DOI:** 10.2174/1874210601812010046

**Published:** 2018-01-31

**Authors:** Amir Reza Rokn, Akram Labibzadeh, Amir Alireza Rasouli Ghohroudi, Ahmad Reza Shamshiri, Somaye Solhjoo

**Affiliations:** Dental Implant Research Center, Tehran University of Medical Sciences, Tehran, Iran.

**Keywords:** Tactile sense, Histomorphomtry, Bone density, Dental implants, Optimal treatment planning, Osseointegration

## Abstract

**Introduction:**

A correct diagnosis and optimal treatment planning is essential for success in implant dentistry. Proper diagnosis of bone quality is an important part of the diagnostic procedure.

**Objective:**

The purpose of this study was to correlate the tactile sense of the surgeon in the assessment of bone density to the histomorphometric analysis of bone quality.

**Methods:**

In this study, 56 bone samples from 33 patients were harvested from implant sites with trephine drills. The samples were analyzed with Image J software. In the samples following parameters were measured: BV/TV, superficial cortical plate thickness, the number and thickness of haversian canals in cortical bone and the number, thickness and distance of trabecules in cancellous bone. The clinical hardness of bone during drilling was evaluated by surgeon according to Misch. GEE analysis with exchangeable correlation structure and linear model was used to evaluate the relationship between the tactile sense of the surgeon and histomorphometric parameters and all analysis was adjusted for two confounding variables: gender and location.

**Results:**

There were 51.79% implants in D2 samples and 48.21% in D3. Bone classification according to Misch was significantly correlated to distance of trabecules in cancellous bone (*P*-value=0.05), and shown marginally significant correlation with mean superficial cortical bone thickness (*P*-value =0.07) and number of haversian canals (*P*-value =0.005) in cortical bone.

**Discussion:**

There were differences between our results and others. The authors believed that these differences mainly are because of confounding factors, that in this study were eliminated. The clinical finding during surgery can approximately explain the histologic properties of bone.

**Conclusion:**

It is concluded that tactile sense of the surgeon can exhibit the histologic properties of the bone, and we are able to estimate the healing prognosis of the bone in implant placement.

## 1 INTRODUCTION

Nowadays, dental implants are a reliable treatment option in edentulous areas of the jaws [1] several properties of osseous tissue can affect implant treatment [2-7]. Primary stability is one of the most important factors for survival rate and it has paramount importance for osseointegration [1, 8-14]. Primary stability is defined as the absence of mobility after placing implant in bone bed [8]. Primary stability is depends on the inserting implant approach [4, 8] quality and quantity of the bone [4, 8, 12], surgical technique and implant geometry (the length, diameter and type) [4, 12].

The term “bone quality” doesn’t have exact definition in literature [5, 8]. Bone quality is consisted of physiologic and structural aspects and shows the degree of bone mineralization [8]. Studies shows that one of the risk factors for implant failure is the low quality of bone [1, 3, 15, 16] and it causes lower treatment predictability [17]. Tactile sense of the surgeon is one of the most popular approaches in determining bone density [18]. Different techniques are available for bone density evaluation: Histomorphometric analysis [1, 18], Tactile sense of the surgeon [7, 10, 11, 19], Dual energy X-ray absorptiometry [1, 20], CT scan [1, 6, 13, 14, 21], Quantitative CT [1, 22], Micro CT [1, 23, 24], CBCT [1, 25], MRI [26], Peak insertion torque value [4, 18, 26], Cutting torque and RFA [4, 26]. Some are suitable before surgery and some are usable during or after implantation. Among these approaches, for example, radiographic evaluations (CT, CBCT, *etc*.) are used before surgery, and other approaches such as assessment of Insertion Torque Value and Resonance Frequency analysis are useful in surgery time [27]. Among these, Radiographic analysis can help us to estimate the treatment planning before surgery, but this requires an extra three-dimensional radiograph [28].

The first and most accepted classification of bone quality introduced with Lekholm & Zarb in 1985 [1, 8, 17, 29]. This classification is based on the amount of trabecular and cortical bone in radiographs in 4 different groups:

Bone quality type I: Almost all areas of the jaws consist of homogeneous and compact bone; Bone quality type II: A thick layer of compact bone surrounding a central area of dense trabecular bone; Bone quality type III: A thin layer of cortical bone around a central area of dense trabecular bone; and Bone quality type IV: A thin layer of cortical bone surrounds a central area of low-density trabecular bone [1, 29]. An important advantage of this classification is that it is possible to use before treatment, therefore it can help the surgeon in treatment planning [1, 30], however, this is a subjective and nonspecific technique and there is a lot of overlaps between its various classifications [18]. Another technique for the assessment of bone quality was introduced by Misch [1, 18]. According to Misch, there are differences in the tactile sense of the surgeon during bed preparation for implantation with different bone qualities [11, 19]. This classification have 4 classes based on the clinical hardness of drilling, consisting of bone type D1 (hardness similar to maple or oak wood), bone type D2 (hardness similar to white pine or spruce wood), bone type D3 (hardness similar to balsa), and bone type D4 (hardness similar to Styrofoam) [1, 11, 19]. This technique is also subjective and requires further studies to rely on [5].

There are several methods for assessment of bone quality, but the documents are rare about the reliability; more studies comparing the gold standard with other methods are required [5]. The best method for assessment of bone micro structure is histomorphometry that is the gold standard method [5, 18]. This is a two-dimensional analysis that is time-consuming [5] and can’t be used in the dental office [18].

The aim of this study was to evaluate the relationship between subjective method of bone density determination; “tactile sense of the surgeon” and the histomorphometric analysis of an implant recipient site biopsies.

## 2 MATERIALS AND METHODS

The samples consisted of 56 bone samples from 33 patients treated with Dyna Dental Engineering BV (Bergenopzoom, the Netherlands) in a private dental office. Eligible samples were selected based on inclusion and exclusion criteria after clinical and radiographic assessments (Table **1**). Written informed consent was obtained from all the selected patients.

**Fig. (1):**
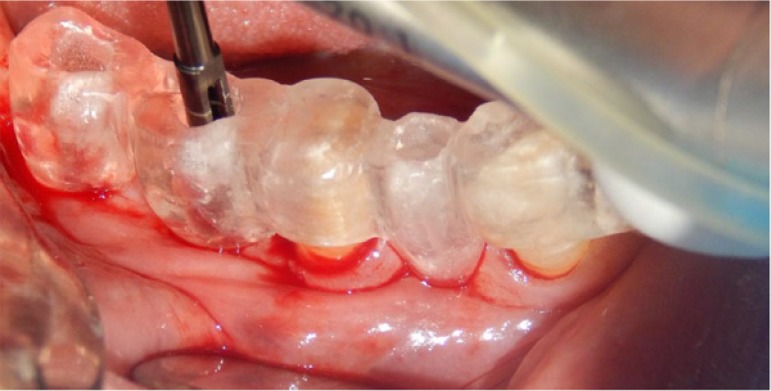
Precise bone sampling using trephine drill and surgical stent.

At the time of surgery after anesthesia a full thickness flap was elevated. For precise drilling a surgical stent was made for each patient. A trephine drill with 2.3 mm inner diameter, 3.0 mm outer diameter and 10 mm height were used as a first drill for implant site preparation. (Fig. **1**) This drill had smaller diameter of the final drill to ensure the correct primary stability. Drilling was made with 8 mm depth and a new drill was used for each patient to have a perfect drilling ability in all patients. Drilling was performed under profuse irrigation at 800 r.p.m. The selection of implant site was randomized. The bone scoring was recorded during site preparation based on Misch (1993). The biopsies were analyzed histomorphometrically in a blind fashion.

### 2.1 Histologic Process

Samples with 3mm diameter were removed from implant site and after rinsing with physiologic solution were fixed in formalin 10%. Then were decalcified with 10% formic acid and then under routine processing prepared in paraffin blocks. Following this processing the samples were longitudinally sectioned in 5 micrometers slices. Three middle sections were selected for histomorphometric analysis and stained with H&E for light microscopic observation. For sample analysis the Image J software was used.

**Fig. (2):**
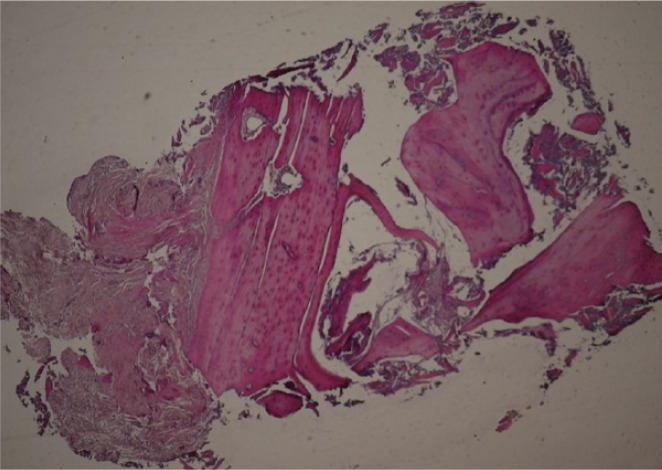
Histologic sample. In measurement of histologic parameters, areas with artifact subtracted from the measures. All the measurements were extracted with the Image J software. In non homogeneous structures the average of data was reported. Cortical plate was defined as the distance from top of the sample to the last distinguishable haversian canal. In all samples, the number and thickness of haversian canals in the cortical bone, bone volume density/ Trabecular bone volume, and trabecular thickness, number and distance was measured.

These following items were assessed in the samples:

1- The thickness of superficial cortical plate
2- Number and thickness of haversian channels in the cortical bone
3- Bone volume density/ Trabecular bone volume (%): It is defined as the coefficient of trabecular bone to tissue volume (Pereira *et al*. 2013)
4- Trabecular thickness (µm): Average of trabecular thickness from min and max
5- Trabecular Number in each mm
6- Trabecular distance (µm): Average of trabecular distance (Fig. **2**)

### 2.2 Statistical Analysis

As in some of the patients more than one implant was inserted, Generalized Estimation Equation analysis with exchangeable correlation structure and linear model was used to evaluate the relationship between the tactile sense of the surgeon and histomorphometric parameters and all analysis was adjusted for two confounding variables: Gender and location.

## 3 RESULTS

All the implanted fixtures reached osseointegration and received prosthesis. All samples had D2 and D3: 29 (51.79%) samples had D2 bone quality and 27 (48.21%) samples had D3. This selection was because of D1 and D4 bone qualities are easy to recognize by tactile sense of the surgeon and the difficulties in practice is for determination between D2 and D3 bone types [18].

Mean BV/TV in D2 was 0.59% (SE= 0.04) and in D3 was 0.49% (SE=0.05). The difference was 0.10% (SE=0.06) and this difference was not statistically significant. (*P*-value =0.13) Mean thickness of superficial cortical plate in D2 was 1481.80 µm(SE= 152.68) and in D3 was 1138.51 µm (SE=120.14). The difference was 343.29 µm (SE=189.17) and this difference was marginally significant. (*P*-value= 0.07) Mean Number of haversian canals in D2 was 23.88 (SE=4.81) and in D3 was 14.67 (SE=3.71). The difference was 9.21 (SE=3.32) and this difference was statistically significant. (*P*-value= 0.05)

Mean thickness of haversian channels in D2 was 167.17 µm(SE=21.69) and in D3 was 268.62 µm (SE=58.57). The difference was 101.45 µm (SE=69.97) and this difference was not significant. (*P*-value= 0.15) Mean trabecular thickness of cancellous bone in D2 was 171.33 µm(SE=10.19) and in D3 was 162.61 µm (SE=12.56). The difference was 8.72 µm (SE=11.95) and this difference was not significant. (*P*-value= 0.47) The Mean trabecular number of cancellous bone in D2 was 20.00 (SE=1.03) and in D3 was 19.33 (SE=1.48). The difference was 0.68 (SE=1.53) and this difference was not significant. (*P*-value= 0.66) Mean trabecular distance of cancellous bone in D2 was 142.87 µm(SE=15.91) and in D3 was 190.70 µm (SE=21.06). The difference was 48.83 µm (SE=24.80) and this difference was marginally significant. (*P*-value= 0.05) (Table **2**).

## 4 DISCUSSION

In this study, we compared two approaches for evaluation of bone density in implant site.

1- Bone quality assessment via evaluation of histomorphometric analysis of bone samples from implanting sites.
2-Bone quality assessment based on a tactile sense of the surgeon according to Misch.The Bone volume assessment is the gold standard in different techniques, but it requires biopsies from implanting bed, and also the results can’t be used in surgery time [27, 28]. Indeed the most popular approach in evaluation of bone quality at the time of implant surgery is the classification of Misch based on the tactile sense of the surgeon. Also, bone assessment based on a tactile sense of the surgeon feedback is the simplest approach [28]. Precise survey of implant recipient site is essential for treatment planning [30, 31]. Therefore, it is necessary for evaluation of the relationship between the gold standard and the simplest approach.

In our study the correlation between the tactile sense of the surgeon and the mean superficial cortical bone thickness, haversian canal number and trabecular distance was statistically significant and the correlation between BV/TV and tactile sense of the surgeon was not statistically significant. It is shown that mean BV/TV in D2 bone quality was higher than D3 bone quality.

Keling *et al*. in 1995 were the first that used the trephine drill for sampling of bone in implant dentistry [31]. After that Trisi and Rao were second that used this approach, they shown that D1 and D4 bone qualities are easier to recognize with tactile sense of the surgeon other than intermediate qualities [18]. Aksoy *et al*. in 2009 also did n’t show any correlation between the density of bone with respect to the tactile sense of the surgeon and quality in BV evaluation [32]. But Trisi, *et al*. suggested that there is significant correlation between the tactile sense of the surgeon and BV/TV [18]. Sennerby, *et al*. in 1992 suggested that the amount of cortical bone that implant passed is one of the most important factors in optimal implant stability [33]. These results are in agreement with ours.

In our study there was a significant correlation between BV/TV and tactile sense of the surgeon before adjusting the confounding factors (gender and location). In other studies the authors didn’t explain any adjustment of these factors, that it can be the main reason for existing differences. In this study, we have shown that as we expect, the denser bone in histomorphometric evaluation is harder on the tactile sense of the surgeon. Indeed, the only parameters that have shown significant correlations with tactile sense of the surgeon was: superficial cortical thickness, haversian canal number of cortical bone and the distance between trabecules of cancellous bone.

Therefore, it can be understood that the tactile sense of the surgeon can exhibit the histologic properties of the bone, and we are able to estimate the healing prognosis of the bone in implant placement. Thus, according to our results we can not exactly explain on the basis of tactile sense that which parameter in histologic evaluation is affecting the results.

## CONCLUSION

Bone quality according to Misch can approximately exhibit the histologic properties of the bone. We can trust on our tactile sense during surgery for estimating the healing process and suitable time for implant loading.

## Figures and Tables

**Table 1:** Inclusion and exclusion criteria.

**Inclusion Criterias**	Completed craniofacial growth
	Healthy and stable periodontium in surgery time
	No systemic contraindication
	Existence of min volume for installing regular implant
**Exclusion Criteria**	Uncompleted craniofacial growth
	Uncontrolled systemic diseases such as diabetes
	Radiotherapy in head and neck region
	Mental and corporeal disabilities
	Active infections
	Simultaneous or staged need for bone augmentation in implant bed

**Table 2:** Results.

	**D2**		**D3**	
	**Mean**	**SD**	**Mean**	**SD**
**Bone volume density/Trabecular bone volume**	0.59%	0.04	0.49%	0.05
**Thickness of superficial cortical plate**	1481.80	152.68	1138.51	120.14
**Number of haversian channels**	23.88	4.81	14.67	3.71
**Thickness of haversian channels**	167.17	21.69	268.62	58.57
**Trabecular thickness of cancellous bone**	171.33	10.19	162.61	12.56
**Trabecular number of cancellous bone**	20.00	1.03	19.33	1.48
**Trabecular distance of cancellous bone**	142.87	15.91	190.70	21.06

## References

[1] Rokn A., Rasouli G. A.A., Daneshmonfared M., Menasheof R., Shamshiri A.R. (2014). Tactile sense of the surgeon in determining bone density when placing dental implant.. Implant Dent..

[2] Fuh L.J., Huang H.L., Chen C.S., Fu K.L., Shen Y.W., Tu M.G., Shen W.C., Hsu J.T. (2010). Variations in bone density at dental implant sites in different regions of the jawbone.. J. Oral Rehabil..

[3] Jaffin R.A., Berman C.L. (1991). The excessive loss of Branemark fixtures in type IV bone: A 5-year analysis.. J. Periodontol..

[4] Linck G.K., Ferreira G.M., De Oliveira R.C., Lindh C., Leles C.R., Ribeiro-Rotta R.F. (2016). The influence of tactile perception on classification of bone tissue at dental implant insertion.. Clin. Implant Dent. Relat. Res..

[5] Pereira A.C., Souza P.P., Souza J.A., Silva T.A., Batista A.C., Ribeiro-Rotta R.F. (2013). Histomorphometrical and molecular evaluation of endosseous dental implants sites in humans: Correlation with clinical and radiographic aspects.. Clin. Oral Implants Res..

[6] Shapurian T., Damoulis P.D., Reiser G.M., Griffin T.J., Rand W.M. (2006). Quantitative evaluation of bone density using the Hounsfield index.. Int. J. Oral Maxillofac. Implants.

[7] Trisi P., Berardi D., Paolantonio M., Spoto G., D’Addona A., Perfetti G. (2013). Primary stability, insertion torque, and bone density of conical implants with internal hexagon: Is there a relationship?. J. Craniofac. Surg..

[8] Marquezan M., Osório A., Sant’Anna E., Souza M.M., Maia L. (2012). Does bone mineral density influence the primary stability of dental implants? A systematic review.. Clin. Oral Implants Res..

[9] Merheb J., Van Assche N., Coucke W., Jacobs R., Naert I., Quirynen M. (2010). Relationship between cortical bone thickness or computerized tomography-derived bone density values and implant stability.. Clin. Oral Implants Res..

[10] Misch C.E. (1988). Editorial comments: The National Institutes of Health Consensus Development Conference statement on dental implants.. Int. J. Oral Implantol..

[11] Misch C.E. (1990). Divisions of available bone in implant dentistry.. Int. J. Oral Implantol..

[12] Nkenke E., Hahn M., Weinzierl K., Radespiel-Tröger M., Neukam F.W., Engelke K. (2003). Implant stability and histomorphometry: A correlation study in human cadavers using stepped cylinder implants.. Clin. Oral Implants Res..

[13] Turkyilmaz I., Tözüm T.F., Tumer C., Ozbek E.N. (2006). Assessment of correlation between computerized tomography values of the bone, and maximum torque and resonance frequency values at dental implant placement.. J. Oral Rehabil..

[14] Turkyilmaz I., Tumer C., Ozbek E.N., Tözüm T.F. (2007). Relations between the bone density values from computerized tomography, and implant stability parameters: A clinical study of 230 regular platform implants.. J. Clin. Periodontol..

[15] Herrmann I., Lekholm U., Holm S., Kultje C. (2005). Evaluation of patient and implant characteristics as potential prognostic factors for oral implant failures.. Int. J. Oral Maxillofac. Implants.

[16] Jemt T., Lekholm U. (1995). Implant treatment in edentulous maxillae: A 5-year follow-up report on patients with different degrees of jaw resorption.. Int. J. Oral Maxillofac. Implants.

[17] Alsaadi G., Quirynen M., Michiels K., Jacobs R., van Steenberghe D. (2007). A biomechanical assessment of the relation between the oral implant stability at insertion and subjective bone quality assessment.. J. Clin. Periodontol..

[18] Trisi P., Rao W. (1999). Bone classification: Clinical-histomorphometric comparison.. Clin. Oral Implants Res..

[19] Misch C.E., Dietsh-Misch F., Hoar J., Beck G., Hazen R., Misch C.M. (1999). A bone quality-based implant system: First year of prosthetic loading.. J. Oral Implantol..

[20] Becker W., Hujoel P.P., Becker B.E., Willingham H. (2000). Osteoporosis and implant failure: An exploratory case-control study.. J. Periodontol..

[21] Norton M.R., Gamble C. (2001). Bone classification: An objective scale of bone density using the computerized tomography scan.. Clin. Oral Implants Res..

[22] Lindh C., Nilsson M., Klinge B., Petersson A. (1996). Quantitative computed tomography of trabecular bone in the mandible.. Dentomaxillofac. Radiol..

[23] Fanuscu M.I., Chang T.L. (2004). Three-dimensional morphometric analysis of human cadaver bone: Microstructural data from maxilla and mandible.. Clin. Oral Implants Res..

[24] Rozé J., Babu S., Saffarzadeh A., Gayet-Delacroix M., Hoornaert A., Layrolle P. (2009). Correlating implant stability to bone structure.. Clin. Oral Implants Res..

[25] Aranyarachkul P., Caruso J., Gantes B., Schulz E., Riggs M., Dus I., Yamada J.M., Crigger M. (2005). Bone density assessments of dental implant sites: 2. Quantitative cone-beam computerized tomography.. Int. J. Oral Maxillofac. Implants.

[26] Taguchi A., Tanimoto K., Akagawa Y., Suei Y., Wada T., Rohlin M. (1997). Trabecular bone pattern of the mandible. Comparison of panoramic radiography with computed tomography.. Dentomaxillofac. Radiol..

[27] Lee S., Gantes B., Riggs M., Crigger M. (2007). Bone density assessments of dental implant sites: 3. Bone quality evaluation during osteotomy and implant placement.. Int. J. Oral Maxillofac. Implants.

[28] Molly L. (2006). Bone density and primary stability in implant therapy.. Clin. Oral Implants Res..

[29] Lekholm U. (1998). Surgical considerations and possible shortcomings of host sites.. J. Prosthet. Dent..

[30] Ribeiro-Rotta R.F., Lindh C., Rohlin M. (2007). Efficacy of clinical methods to assess jawbone tissue prior to and during endosseous dental implant placement: A systematic literature review.. Int. J. Oral Maxillofac. Implants.

[31] Klinge B., Johansson C., Albrektsson T., Hallström H., Engdahl T. (1995). A new method to obtain bone biopsies at implant sites peri-operatively: technique and bone structure.. Clin. Oral Implants Res..

[32] Aksoy U., Eratalay K., Tözüm T.F. (2009). The possible association among bone density values, resonance frequency measurements, tactile sense, and histomorphometric evaluations of dental implant osteotomy sites: A preliminary study.. Implant Dent..

[33] Sennerby L., Thomsen P., Ericson L.E. (1992). A morphometric and biomechanic comparison of titanium implants inserted in rabbit cortical and cancellous bone.. Int. J. Oral Maxillofac. Implants.

